# Supplementary material for: Know-do gaps for cardiovascular disease care in Cambodia: Evidence on clinician knowledge and delivery of evidence-based prevention actions
Source: PLOS Glob Public Health. 2022 Sep 1;2(9):e0000862. doi: 10.1371/journal.pgph.0000862 (PMC10022025; doi:10.1371/journal.pgph.0000862)
Supplement: S1 Text — (DOCX) [file pgph.0000862.s001.docx]

**S1 Text. Relationship between equipment availability and blood glucose screening**

24 out of 114 facilities had the necessary equipment to conduct a blood glucose measurement (both a glucometer and test strips). Using univariate Poisson and Linear Probability Models with an indicator for equipment availability as predictor and an indicator for blood glucose measurement as the dependent variable, we found some evidence that the diabetes care gaps we describe in the main manuscript differ depending on the access to functioning glucometers and test strips, although this result is estimated with very wide confidence intervals that overlap the null. The estimates rely on 190 observed patient consultations. We clustered standard errors at the health facility level.

**Table A.** Association between the availability of diabetes equipment and the probability of blood glucose measurements based on Linear Probability and Poisson regression models

| **Model** | **Sample** | **Estimate** | **95% Confidence**  Lower bound | **95% Confidence**  Upper bound |
| --- | --- | --- | --- | --- |
|  |  |  |  |  |
| Linear Probability Model | Patient observations | Prevalence difference: 0.11 | -0.14 | 0.36 |
|  |  |  |  |  |
| Poisson Regression Model | Patient observations | Prevalence ratio: 5.27 | 0.9 | 30.92 |
