# Supplementary material for: Know-do gaps for cardiovascular disease care in Cambodia: Evidence on clinician knowledge and delivery of evidence-based prevention actions
Source: PLOS Glob Public Health. 2022 Sep 1;2(9):e0000862. doi: 10.1371/journal.pgph.0000862 (PMC10022025; doi:10.1371/journal.pgph.0000862)
Supplement: S2 Text — (DOCX) [file pgph.0000862.s002.docx]

**S2 Text. Detailed regression results for main study figures**

All Poisson regression models were estimated based on 190 patient observations. Standard errors were clustered at the health facility level.

**Differences in cardiovascular disease care across clinician cadres**

We estimated the following models:

$Outcome= \beta_{0}+\beta_{1}*Midwife+\beta_{2}*Nurse$,

where outcome is one of the eight considered measurement and risk assessment items (measured blood pressure once, measured blood pressure at least twice, measured blood glucose, measured body mass index, asked about smoking, asked about alcohol consumption, asked about diet, and asked about physical activity).

**Table B** Regression results for outcome: blood pressure measured once

| **Independent Variable** | **Coefficient** | **SE** | **95% Confidence**  Lower bound | **95% Confidence**  Upper bound | **p-value** |
| --- | --- | --- | --- | --- | --- |
| Intercept | -0.1272 | 0.1071 | -0.3372 | 0.0828 | 0.2353 |
| Midwife | -0.3605 | 0.2023 | -0.7571 | 0.036 | 0.0748 |
| Nurse | -0.1079 | 0.1224 | -0.3478 | 0.132 | 0.3779 |

**Table C** Regression results for outcome: blood pressure measured twice

| **Independent Variable** | **Coefficient** | **SE** | **95% Confidence**  Lower bound | **95% Confidence**  Upper bound | **p-value** |
| --- | --- | --- | --- | --- | --- |
| Intercept | -2.6177 | 0.6353 | -3.8629 | -1.3725 | < 0.001 |
| Midwife | 0.1054 | 0.9242 | -1.706 | 1.9168 | 0.9092 |
| Nurse | 0.1017 | 0.7186 | -1.3067 | 1.5101 | 0.8875 |

**Table D** Regression results for outcome: blood glucose measured

| **Independent Variable** | **Coefficient** | **SE** | **95% Confidence**  Lower bound | **95% Confidence**  Upper bound | **p-value** |
| --- | --- | --- | --- | --- | --- |
| Intercept | -1.748 | 0.6401 | -3.0026 | -0.4933 | 0.0063 |
| Midwife | -18.5546 | 0.6821 | -19.8915 | -17.2178 | < 0.001 |
| Nurse | -1.5609 | 0.8777 | -3.2812 | 0.1593 | 0.0753 |

**Table E** Regression results for outcome: BMI measured

| **Independent Variable** | **Coefficient** | **SE** | **95% Confidence**  Lower bound | **95% Confidence**  Upper bound | **p-value** |
| --- | --- | --- | --- | --- | --- |
| Intercept | -1.9021 | 0.6516 | -3.1793 | -0.6249 | 0.0035 |
| Midwife | -0.4279 | 0.9546 | -2.2988 | 1.443 | 0.654 |
| Nurse | -1.3218 | 0.8561 | -2.9998 | 0.3562 | 0.1226 |

**Table F** Regression results for outcome: asked about diet

| **Independent Variable** | **Coefficient** | **SE** | **95% Confidence**  Lower bound | **95% Confidence**  Upper bound | **p-value** |
| --- | --- | --- | --- | --- | --- |
| Intercept | -0.6212 | 0.2152 | -1.0429 | -0.1994 | 0.0039 |
| Midwife | -0.279 | 0.3512 | -0.9673 | 0.4092 | 0.4269 |
| Nurse | -0.4214 | 0.2602 | -0.9314 | 0.0886 | 0.1053 |

**Table G** Regression results for outcome: asked about physical activity

| **Independent Variable** | **Coefficient** | **SE** | **95% Confidence**  Lower bound | **95% Confidence**  Upper bound | **p-value** |
| --- | --- | --- | --- | --- | --- |
| Intercept | -1.1979 | 0.3845 | -1.9516 | -0.4443 | 0.0018 |
| Midwife | -1.6806 | 0.7191 | -3.0901 | -0.2712 | 0.0194 |
| Nurse | -1.0977 | 0.5097 | -2.0968 | -0.0986 | 0.0313 |

**Table H** Regression results for outcome: asked about smoking

| **Independent Variable** | **Coefficient** | **SE** | **95% Confidence**  Lower bound | **95% Confidence**  Upper bound | **p-value** |
| --- | --- | --- | --- | --- | --- |
| Intercept | -1.8068 | 0.5898 | -2.9628 | -0.6508 | 0.0022 |
| Midwife | -0.0742 | 0.7902 | -1.623 | 1.4745 | 0.9252 |
| Nurse | 0.1469 | 0.6363 | -1.1002 | 1.3939 | 0.8174 |

**Table I** Regression results for outcome: asked about alcohol consumption

| **Independent Variable** | **Coefficient** | **SE** | **95% Confidence**  Lower bound | **95% Confidence**  Upper bound | **p-value** |
| --- | --- | --- | --- | --- | --- |
| Intercept | -1.9711 | 0.7017 | -3.3465 | -0.5957 | 0.005 |
| Midwife | 0.1912 | 0.8775 | -1.5287 | 1.911 | 0.8275 |
| Nurse | 0.4696 | 0.7306 | -0.9624 | 1.9016 | 0.5204 |

**Association between cardiovascular disease care and years of experience**

We estimated the following models:

$Outcome= \beta_{0}+\beta_{1}*Experience$,

where outcome is one of the eight considered measurement and risk assessment items (measured blood pressure once, measured blood pressure at least twice, measured blood glucose, measured body mass index, asked about smoking, asked about alcohol consumption, asked about diet, and asked about physical activity) and Experience is a continuous measure of years practicing medicine.

**Table J** Regression results for outcome: blood pressure measured once

| **Independent Variable** | **Coefficient** | **SE** | **95% Confidence**  Lower bound | **95% Confidence**  Upper bound | **p-value** |
| --- | --- | --- | --- | --- | --- |
| Intercept | -0.1796 | 0.0776 | -0.3317 | -0.0274 | 0.0207 |
| Experience | -0.0072 | 0.0061 | -0.0191 | 0.0047 | 0.2331 |

**Table K** Regression results for outcome: blood pressure measured twice

| **Independent Variable** | **Coefficient** | **SE** | **95% Confidence**  Lower bound | **95% Confidence**  Upper bound | **p-value** |
| --- | --- | --- | --- | --- | --- |
| Intercept | -2.1865 | 0.471 | -3.1096 | -1.2634 | < 0.001 |
| Experience | -0.0324 | 0.0392 | -0.1093 | 0.0445 | 0.4085 |

**Table L** Regression results for outcome: blood glucose measured

| **Independent Variable** | **Coefficient** | **SE** | **95% Confidence**  Lower bound | **95% Confidence**  Upper bound | **p-value** |
| --- | --- | --- | --- | --- | --- |
| Intercept | -2.7754 | 0.6232 | -3.9968 | -1.554 | < 0.001 |
| Experience | -0.0272 | 0.0355 | -0.0969 | 0.0424 | 0.4436 |

**Table M** Regression results for outcome: BMI measured

| **Independent Variable** | **Coefficient** | **SE** | **95% Confidence**  Lower bound | **95% Confidence**  Upper bound | **p-value** |
| --- | --- | --- | --- | --- | --- |
| Intercept | -3.332 | 0.5099 | -4.3315 | -2.3326 | < 0.001 |
| Experience | 0.0412 | 0.0255 | -0.0088 | 0.0912 | 0.1059 |

**Table N** Regression results for outcome: asked about diet

| **Independent Variable** | **Coefficient** | **SE** | **95% Confidence**  Lower bound | **95% Confidence**  Upper bound | **p-value** |
| --- | --- | --- | --- | --- | --- |
| Intercept | -1.1533 | 0.1954 | -1.5362 | -0.7704 | < 0.001 |
| Experience | 0.0157 | 0.0105 | -0.0049 | 0.0362 | 0.1352 |

**Table O** Regression results for outcome: asked about physical activity

| **Independent Variable** | **Coefficient** | **SE** | **95% Confidence**  Lower bound | **95% Confidence**  Upper bound | **p-value** |
| --- | --- | --- | --- | --- | --- |
| Intercept | -2.3366 | 0.4005 | -3.1215 | -1.5517 | < 0.001 |
| Experience | 0.015 | 0.023 | -0.03 | 0.0601 | 0.5137 |

**Table P** Regression results for outcome: asked about smoking

| **Independent Variable** | **Coefficient** | **SE** | **95% Confidence**  Lower bound | **95% Confidence**  Upper bound | **p-value** |
| --- | --- | --- | --- | --- | --- |
| Intercept | -2.0193 | 0.3076 | -2.6221 | -1.4165 | < 0.001 |
| Experience | 0.0231 | 0.0162 | -0.0087 | 0.0549 | 0.155 |

**Table Q** Regression results for outcome: asked about alcohol consumption

| **Independent Variable** | **Coefficient** | **SE** | **95% Confidence**  Lower bound | **95% Confidence**  Upper bound | **p-value** |
| --- | --- | --- | --- | --- | --- |
| Intercept | -1.7488 | 0.2941 | -2.3253 | -1.1724 | < 0.001 |
| Experience | 0.012 | 0.0155 | -0.0183 | 0.0424 | 0.4372 |

$$Blood glucose measured= \beta_{0}+\beta_{1}*Equipment$$
