# Supplementary material for: Know-do gaps for cardiovascular disease care in Cambodia: Evidence on clinician knowledge and delivery of evidence-based prevention actions
Source: PLOS Glob Public Health. 2022 Sep 1;2(9):e0000862. doi: 10.1371/journal.pgph.0000862 (PMC10022025; doi:10.1371/journal.pgph.0000862)
Supplement: Attachment — Submitted filename: response letter.pdf [file pgph.0000862.s004.pdf]

Editorial Comments

1. Please amend your current ethics statement to address the following concerns: Please explain how you recorded/documented participant consent, and if the ethics committees/IRBs approved this consent procedure.

   We have revised our manuscript to include this information at the end of our Methods section (page 10, new text in bold):

   > We obtained written informed consent from health administrators to collect data within health centres and verbal informed consent from clinicians and patients before collecting any data. We used verbal, rather than written consent, to avoid disrupting regular clinical services and to avoid literacy and comprehension challenges with patients. **Participant consent was noted by the observer in the data collection form. This procedure was approved by the ethics committees.**

2. Please include a complete copy of PLOS' questionnaire on inclusivity in global research in your revised manuscript.

   We have completed the Inclusivity in Global Research questionnaire and have uploaded it with our revised submission.

3. Please update the completed 'Competing Interests' statement, including any COIs declared by your co-authors. If you have no competing interests to declare, please state "The authors have declared that no competing interests exist".

   We have updated the Competing Interests statement to be clear that we declare no competing interests (page 16).

4. In the online submission form, you indicated that your data will be submitted to a repository upon acceptance. We strongly recommend all authors deposit their data before acceptance, as the process can be lengthy and hold up publication timelines. Please note that, though access restrictions are acceptable now, your entire data will need to be made freely accessible if your manuscript is accepted for publication. This policy applies to all data except where public deposition would breach compliance with the protocol approved by your research ethics board. If you are unable to adhere to our open data policy, please kindly revise your statement to explain your reasoning and we will seek the editor's input on an exemption. Please be assured that, once you have provided your new statement, the assessment of your exemption will not hold up the peer-review process.

   We are in the final steps of preparing our data for online submission and anticipate uploading it within the next two weeks.

5. Please amend your detailed Financial Disclosure statement. This is published with the article, therefore should be completed in full sentences and contain the exact wording you wish to be published.

   We have corrected our Financial Disclosure statement.

Reviewer 1

1. Study population: The title, abstract and introduction mentions "Clinician" as the study population. However, the result section mentions only 10% of the case vignettes and patients observations are really from clinician. The authors should not misguide the readers in this regard.

   We used the term clinician, rather than for example "physician," specifically since it encompasses all individuals that provide clinical care, including nurses and midwives. However, to prevent misunderstandings and since our aim was not to misguide readers, we have now added a sentence to the Methods section (page 7) specifically clarifying our use of "clinician":

   > "We use the standard definition of "clinician" to mean all individuals who provide clinical patient care. In the Health Centres in Cambodia, this includes midwives, nurses, and physicians/medical doctors."

2. There is no mention about the basic demographic and clinical details of the patients who have been observed as part of consultations.

   We only collected information on the age and sex of the patients being observed. We made this decision for two main reasons:

   1. *As per national guidelines, screening actions should be happening for all adult patients and not just those with certain clinical presentations or histories. For this reason, we felt it was appropriate to evaluate clinical behavior among a general all-adult patient pool.*
   2. *We wanted to minimize clinical disruptions and not interfere with patient care; for this reason, we felt it was best to only collect basic information rather than administer a detailed questionnaire.*

   While this focus on the general adult patient pool would not produce a biased assessment of screening actions (since they should happen for all adult patients), it does not allow us to investigate whether some patients were more likely to receive screening actions compared to others. This is an important area for future work and we have revised the manuscript to mention this in the limitations section (page 15):

   > "Lastly, our study did not investigate whether some types of patients were more likely to receive screening actions compared to others. This is an important question and future research should collect more detailed information on patient characteristics to determine whether certain population groups are being disproportionately overlooked by clinician screening."

3. No details available on how many different types of clinical vignettes were used.

We thank the reviewer for catching this omission. We have revised our manuscript (page 8) to be clear that we used one clinical vignette.

4. Further, related to "Adherence to evidence-based prevention guidelines", neither it was not defined by the authors nor it was comprehensive assessment. Like a) whether history of pre-existing diabetes and hypertension and its treatment status was asked by the doctor, nurse or midwife; and presence of any previous episode of CVD are missing.

   We thank the reviewer for noting that we do not measure all aspects of CVD preventive care. We have revised the manuscript throughout (including the title and abstract) to remove mention of "adherence to guidelines" and rather be clearer that we are measuring clinical behavior for a set of clinical actions but not necessarily adherence to the entire set of guideline actions. We also now acknowledge this point in the limitations section, but note that since CVD screening actions are not substitutes for one another, omitting one action (such as asking about family history) would not bias our estimate of how frequently the other care actions are being performed (page 15):

   > "While we measure many key aspects of preventive care, our analysis does not examine all actions, including for example an assessment of family history. However, as family history is not a substitute for the other actions, this omission would not bias our assessment of the share of clinicians that are not meeting the other clinical actions."

5. I could not understand and also not agreeing with the authors on comparing the midwifes and nurses with doctors who have different knowledge and skill levels and also care responsibilities.

   We focused on all those providing clinical care in the health centres, including midwives and nurses. We believe this focus on all clinicians and not just physicians was especially relevant since the majority of care at the health facility level is provided by nurses and midwives, rather than physicians. The nurses and midwives also have similar care responsibilities regarding CVD risk assessments and should be conducting the basic screening actions we examine in the manuscript. However, to the reviewer's concern, we also stratified our results by nurses, midwives, and physicians (Figure 2). Although we observe small differences, we do not find evidence that care meaningfully differs across these groups (page 12):

   > "We did not find strong evidence of differences in the size of CVD care gaps by clinician cadre (**Fig 2**). The only exception was that midwives (RR: 0.19 [0.056, 0.760]) and nurses (RR: 0.33 [0.122, 0.906]) were less likely to ask patients about their physical activity (RR: 0.19 [0.056, 0.760]) and collect a first blood pressure measurement (RR: 0.83 [0.70, 0.98]) compared to physicians."

6. What services are provided routinely in the study health centres of Cambodia;

   We thank the reviewer for noting this omission in the description of the study sites and have revised our manuscript (pages 5-6) to include this information:

   > "The Health Centres provide a range of basic preventive and curative services including care for infectious and acute conditions (like malaria, respiratory disease, and tuberculosis), family planning and ante and postnatal care, and basic care and screening for chronic non-communicable diseases including screening for hypertension and diabetes. The full range of services that the Health Centres provide is specified in the Cambodian Minimum Package of Activities."

7. What are the brief job responsibilities of midwifes, nurses and doctors, e.g if Midwife or nurse is only responsible for providing counselling service, then of course she will not test the patient for blood sugar;

   We thank the reviewer for raising this important point. Nurses and midwives are indeed expected to provide the basic CVD screening actions we investigate in our manuscript. We have revised our manuscript in the Setting section (page 6) to include this point and provide more detail on the responsibilities of the different types of clinicians:

   > "Clinical care in the Health Centres is provided by nurses, midwives, and medical doctors (physicians), with nurses and midwives providing the majority of care. While some of the care responsibilities are differentiated by clinician cadre (e.g. midwives focus more heavily on ante and postnatal care while physicians focus more heavily on the outpatient department), there is substantial overlap in the care responsibilities by cadre, and importantly, all three clinician types are expected to provide CVD preventive care that includes screening for hypertension and diabetes and assessing a range of behavioral risk factors."

8. Availability of blood sugar machine, sphygmomanometer, stadiometer and weighing machine.

   We thank the reviewer for raising this important question and omission from our initial manuscript. All facilities we visited had a scale and height board and either a manual or electronic sphygmomanometer. Only a minority of facilities had a glucometer and test strips. We found some evidence that having a glucometer and test strips were associated with a greater chance that clinicians measured blood sugar; however, this estimate had a wide confidence interval that overlapped the null and the proportion of visits that received a blood glucose measurement within facilities with this equipment was still low. We have revised our manuscript to

include this information (page 13; the results are now presented in the Supporting Information):

> "An important question is whether the low shares of BMI, blood glucose, and blood pressure measurement is due to clinicians not having access to measurement devices. However, every facility in our study reported having a scale and height board and a sphygmomanometer, making this explanation unlikely for BMI and blood pressure. We did find that only 21% of facilities had access to glucometers and test strips and that clinicians in facilities with these materials were 11 percentage points more likely to measure blood glucose although the 95% confidence interval for this estimate was wide and overlapped the null. Importantly, even among facilities with access to blood glucose measurement devices, just 14% of adult patients had their blood pressure screened, indicating a large remaining gap that is not attributable to equipment availability (Supporting Information)."

9. The overall and DM/HTN/CVD patient load, the % of acute and non-acute visits

   We have revised the manuscript in the methods section to now provide information on the overall patient caseloads in the selected facilities. Specifically, we included (page 7):

   > "The median number of patients seen in the facilities per month was 773, ranging from a minimum of 114 to a maximum of 2820 patients."

   Unfortunately, we did not have the information on what proportion of these patients had diabetes mellitus, hypertension, or cardiovascular disease. However, since the clinical actions we examine are all relevant to the screening of non-diagnosed patients, the focus of our manuscript is on all patients and not just those that are already diagnosed with diabetes mellitus, hypertension, or cardiovascular disease.

10. Availability of any national or local guidelines on prevention and control of CVDs, its implementation and training status;

    We have revised the manuscript in the Setting section to detail the national guidelines, providing citations to the actual guideline documents (pages 6-7):

    > "The Government of Cambodia established evidence-based care guidelines for cardiovascular disease prevention (in the form of guidelines for nutrition, physical activity, hypertension, and diabetes care) as part of the first National Plan for Prevention and Control of Non-Communicable Diseases 2007-2010. These guidelines were updated for the second, 2013-2020, and third, 2018-2077 plans."

We have also noted in the Setting and Discussion section that there have been no assessments of the training status on these guidelines.

In the Setting section (page 7):

> Unfortunately, there is no definitive evidence on how thoroughly clinicians in Health Centres were trained on these guidelines; this is an important point we return to when contextualizing our findings in the Discussion section.

In the Discussion section (pages 13-14):

> Our results implicate several areas where clinical knowledge improvements are needed. While the care actions we study are all outlined in the official Cambodian care guidelines, there is limited evidence on whether clinicians practicing in health facilities are aware of and have been trained on these guideline actions. Therefore, measuring what share of clinicians have been trained on guideline actions and subsequently, improving clinician training are likely an important first step for improving clinical care in the country.

11. What was the flow of observed patients in the study health centre. Whether all patients goes through everyone or meets only certain people. It is also not clear whether the same patient consultation was observed in all three places and counted as one or three?

We have revised the manuscript in the Setting section (page 6) to describe the flow of patients:

> "Patients that arrive at the Health Centres are first directed to a reception area, after which they are directed to either an outpatient department for general medicine (primary care) or to a maternal child health/delivery department. After their consultation, patients are then directed to the station for wound dressing (if needed following minor surgeries) and pharmacy. If no additional care is required at that time the patient is then sent home; otherwise, the patient is admitted and referred to a public Referral Hospital."

We observed distinct patients in each facility (e.g. it was not just the case that the same patient met a nurse, midwife, and physician and was thus counted as three observations). We have revised the manuscript in the Methods section to make this clearer (page 8):

> "We observed a unique set of patients in each facility (e.g. it was not the case that the same patient met a nurse, midwife, and physician and was counted as three observations)."

12. Whether the care is Paid or unpaid or free for patient?

We have revised our manuscript in the Setting section to now include the following information (page 6):

> "Care in the Health Centres is free for poorer families through Cambodia's Health Equity Fund; families that are not eligible for the Health Equity Fund have to pay for their care based on a pre-specified fee schedule."

13. Unfortunately, the authors are also made discussion points on information which is not part of their results. Like Line number 191-192 of page 11, "First, they suggest that individuals with diabetes are being overlooked and left undiagnosed by the public primary care system in Cambodia.". In the current manuscript, other than testing for blood sugar, the study did not explore anything at all to bring this point for discussion.

We have revised this sentence to now say:

> "Due to the low rates of blood sugar measurement, it is likely that many individuals with diabetes are not being detected."

We believe this conclusion is justified and would like to respectfully disagree with the reviewer's statement that "the study did not explore at all to bring this point for discussion" since blood sugar measurement is the key necessary condition for diabetes diagnosis.

14. Similarly, without testing any educational intervention, the authors are discussing its effectiveness.

We thank the reviewer for raising this concern. As part of the discussion section, we discussed whether educational interventions alone will be able to close the gaps we observe, citing several prior studies:

> 22. Chauhan BF, Jeyaraman MM, Mann AS, Lys J, Skidmore B, Sibley KM, et al. Behavior change interventions and policies influencing primary healthcare professionals' practice-an overview of reviews. Implement Sci. 2017; 12:3. Epub 2017/01/05. doi: 10.1186/s13012-016-0538-8 PMID: 28057024.
> 23. Seidu S, Walker NS, Bodicoat DH, Davies MJ, Khunti K. A systematic review of interventions targeting primary care or community based professionals on cardio-metabolic risk factor control in people with diabetes. Diabetes Res Clin Pract. 2016; 113:1–13. Epub 2016/01/21. doi: 10.1016/j.diabres.2016.01.022 PMID: 26972954.

24. Mostofian F, Ruban C, Simunovic N, Bhandari M. Changing physician behavior: what works. Am J Manag Care. 2015; 21:75–84.
25. Thepwongsa I, Kirby C, Schattner P, Shaw J, Piterman L. Type 2 diabetes continuing medical education for general practitioners: what works? A systematic review. Diabet Med. 2014; 31:1488–97. doi: 10.1111/dme.12552 PMID: 25047877.
26. Judson TJ, Volpp KG, Detsky AS. Harnessing the Right Combination of Extrinsic and Intrinsic Motivation to Change Physician Behavior. JAMA. 2015; 314:2233–4. doi: 10.1001/jama.2015.15015 PMID: 26624821.
27. Shah MK, Kondal D, Patel SA, Singh K, Devarajan R, Shivashankar R, et al. Effect of a multicomponent intervention on achievement and improvements in quality-of-care indices among people with Type 2 diabetes in South Asia: the CARRS trial. Diabet Med. 2020; 37:1825–31. Epub 2019/09/28. doi: 10.1111/dme.14124 PMID: 31479537

As is common and accepted practice in Discussion sections, we contextualized our findings with respect to the prior literature with citations to the respective peer-reviewed articles.

15. Line 206: Angina is part of CVD and hence it cannot be counted in other clinical areas.

    We have removed the mention of angina from this sentence.

Reviewer 2

Appreciate the authors for choosing an important, relevant topic, the missed opportunities for screening for cardiovascular risk factors in resource limited primary health care setting. The article is well written and the study was conducted following appropriate methodology and tools. This will be interesting for the readers and managers of health programmes. To enrich the article the following may be considered:

1. Section: Abstract: line 28 mentions: "only 2.3% (0.9%, 5.6%) their BMI measured" Instead of "BMI" it should be Blood Glucose.

   We thank the reviewer for noting this error and have updated the manuscript to fix it.

2. The year of conduction of the study and period of data collection was not mentioned.

   We have updated the methods section (page 7) to include this information:

   > "We use data collected from 114 public primary Health Centres across Cambodia between July and September 2020."

3. Two different age groups (>30 and =40 years) were selected for mentioned data collection tools (direct observation and responses following vignettes). The authors may clarify the reasons for having separate "age" criteria and if this age group selection (if all observed patients were relatively younger) could have affected the result of the study.

   We thank the reviewer for catching this important discrepancy in our initial submission. We have revised the results throughout the manuscript to be consistent and focus only on observations for patients ages 40 and above. This change did not affect any of our conclusions and our main findings remain consistent. The exact numerical results changed slightly due to the change in sample size and composition, however.

4. Section: Statistical Analysis: line 135: "We examined the correlation with years of experience using similar regression models..." The word correlation may be replaced with association

   As per the reviewer's suggestion, we have replaced the word "correlation" with "association" throughout the paper.
