# Supplementary material for: Know-do gaps for cardiovascular disease care in Cambodia: Evidence on clinician knowledge and delivery of evidence-based prevention actions
Source: PLOS Glob Public Health. 2022 Sep 1;2(9):e0000862. doi: 10.1371/journal.pgph.0000862 (PMC10022025; doi:10.1371/journal.pgph.0000862)
Supplement: Attachment — Submitted filename: Response letter.pdf [file pgph.0000862.s005.pdf]

27 May 2022

Dear Dr. Tromp:

Thank you for the opportunity to revise and resubmit our manuscript for possible publication in *Plos Global Public Health*.

We have listed the requested revisions below with our responses in italics. Where appropriate, we have directly copied in the revised and/or relevant material.

We hope the revised manuscript will be of interest to *Plos Global Public Health*. Please do not hesitate to contact us if you have any additional questions or would like us to make any additional changes.

Best regards,

Nikkil Sudharsanan

Editorial Comments

1. Please provide an Author Summary. This should appear in your manuscript between the Abstract (if applicable) and the Introduction, and should be 150–200 words long. The aim should be to make your findings accessible to a wide audience that includes both scientists and non-scientists.

   We have revised our manuscript to include an Author Summary, copied here:

   > Cardiovascular diseases (CVDs) are a major and growing cause of mortality in Cambodia. Primary-care clinicians play an important role in preventing CVDs: through screening, clinicians can identify which patients need care for hypertension and diabetes, and through risk assessments, which patients need to make important behavior changes. Although Cambodian care guidelines recommend that clinicians conduct these screenings and assessments for all adult patients regardless of what they sought care for, there have been no assessments of whether patients receive these essential preventive care actions. We collected data from 114 public primary healthcare facilities across Cambodia to investigate this question. We found evidence of large clinical care shortfalls for several CVD care actions. Less than 10% of patients had their body mass index or blood glucose measured and less than 21% were asked about their health behaviors. While we found that some of these shortfalls were related to clinician knowledge gaps, many were related to gaps between what clinicians knew and how they provided care. Our results suggest that there is substantial CVD prevention potential being lost in Cambodia and that efforts to improve preventive care should target both clinical knowledge and the bottlenecks between knowledge and care behavior.

2. Please review your reference list to ensure that it is complete and correct. If you have cited papers that have been retracted, please include the rationale for doing so in the manuscript text, or remove these references and replace them with relevant current references. Any changes to the reference list should be mentioned in the rebuttal letter that accompanies your revised manuscript. If you need to cite a retracted article, indicate the article's retracted status in the References list and also include a citation and full reference for the retraction notice.

   We confirm that our reference list is complete and correct and that no cited papers have been retracted.

3. Table 1. The column title (Vignettes and patient observations) not matching with row title (Years practicing).

We apologize for the unclear labeling in this table. The row title of "Years practicing" refers to the clinicians that are part of the vignette and patient observation sample. We have revised the labels in this table to make this clearer:

Table 1. Descriptive characteristics of the sampled clinicians

	**Clinicians who responded to the clinical vignettes (N=337)**	**Clinicians providing care to the patients whose consultations were observed (N=190)**
**Qualification of clinicians in each sample**		
medical doctor	34 (10.1%)	28 (14.7%)
midwife	152 (45.1%)	33 (17.4%)
nurse	151 (44.8%)	129 (67.9%)
**Years practicing among clinicians in each sample**		
Mean (SD)	10.8 (9.22)	12.1 (9.89)
Median [Min, Max]	8.00 [0, 39.0]	8.00 [0, 34.0]

4. Results: Paragraph 1: Patient observation percentages (by nurses) need to be changed.

   We thank the editor for catching this error and have corrected it in the revised manuscript.

5. Though prevalence ratio is correctly mentioned in figures, the same need to be updated in results text (instead of RR).

   We thank the editor for catching this error and have corrected it in the revised manuscript.
